# The copper(II)-binding tripeptide GHK, a valuable crystallization and phasing tag for macromolecular crystallography

**DOI:** 10.1107/S2059798320013741

**Published:** 2020-11-19

**Authors:** Alexander Mehr, Fabian Henneberg, Ashwin Chari, Dirk Görlich, Trevor Huyton

**Affiliations:** a Department of Structural Dynamics, Max Planck Institute for Biophysical Chemistry, Göttingen, Germany; b Department of Cellular Logistics, Max Planck Institute for Biophysical Chemistry, Göttingen, Germany

**Keywords:** phasing, crystallization, GHK, SAD

## Abstract

The growth of diffraction-quality crystals and experimental phasing remain two of the main bottlenecks in protein crystallography. Here, the high-affinity copper(II)-binding tripeptide GHK was fused to the N-terminus of a GFP variant and an MBP-FG peptide fusion. The GHK tag promoted crystallization, with various residues (His, Asp, His/Pro) from symmetry molecules completing the copper(II) square-pyramidal coordination sphere. Rapid structure determination by copper SAD phasing could be achieved, even at a very low Bijvoet ratio or after significant radiation damage. When collecting highly redundant data at a wavelength close to the copper absorption edge, residual S-atom positions could also be located in log-likelihood-gradient maps and used to improve the phases. The GHK copper SAD method provides a convenient way of both crystallizing and phasing macromolecular structures, and will complement the current trend towards native sulfur SAD and MR-SAD phasing.

## Abstract 2

A novel three-residue tag containing the residues GHK that can be used to promote crystallization and in SAD phasing experiments using its tightly bound copper ion is described.

## 1. Introduction   

In an X-ray diffraction experiment, information relating to the phase of each reflection is lost, which poses the so-called ‘phase problem’ in protein crystallography. These phase estimations can then be obtained by molecular replacement (MR), where a known homologous structure is positioned in the new unit cell and phases are calculated from this model. Alternatively, experimental phasing approaches that rely on finding the positions of heavy atoms or anomalous scatterers are used to obtain the phase information (Taylor, 2010 ▸).

In recent decades, tremendous advances have been made in protein crystallography in hardware, software, methodology and data-collection methods. In the crystallization field, alternative precipitant screens (Grimm *et al.*, 2010 ▸) or combinations such as in the BCS or Morpheus screens (Chaikuad *et al.*, 2015 ▸; Gorrec, 2009 ▸) are helping to increase crystallization space. To complement this, high-throughput robotics, mixed matrix seeding and automatic crystal detection are only some of the methods being used or developed to reduce the crystallization bottleneck (D’Arcy *et al.*, 2014 ▸; Dierks *et al.*, 2010 ▸). Keeping track of all of this information from thousands of screening data sets is also no longer only the domain of industrial laboratories, and extensive database mining and sharing of data is critical for the success of many academic crystallization facilities (Newman *et al.*, 2012 ▸). For structure solution, structural genomics has provided the Protein Data Bank (PDB) with a large number of deposited structures that can now be used in MR searches via automated pipelines (*MrBUMP*, *BALBES*, *MORDA*; Keegan *et al.*, 2018 ▸; Long *et al.*, 2008 ▸; Vagin & Lebedev, 2015 ▸). These software packages offer a ‘brute-force’ screening approach and often give solutions even for very low homology structures. However, even good search models can fail to produce refinable solutions, particularly in cases with partial search models comprising a fraction of the asymmetric unit or where conformational changes in the protein complicate the search procedure. In these cases, experimental phasing methods are still necessary for structure solution. Solving lower resolution structures with data at worse than 3.5 Å resolution still represents a considerable challenge for MR since a low data:parameter ratio combined with potential model bias can lead to difficulties in the building and refinement process. In such cases, combining additional information from anomalously scattering atoms using MR-SAD can be used to improve phases and to rebuild with minimized model bias (Panjikar *et al.*, 2009 ▸; Read & McCoy, 2011 ▸; Schuermann & Tanner, 2003 ▸; Skubák *et al.*, 2018 ▸).

For *de novo* structure determination, anomalous diffraction methods that exploit the breakdown of Friedel’s law at the X-ray absorption edge of an anomalous scatterer have become the preferred method of structure determination. The strength of the signal depends upon the properties of the sample, including the crystal symmetry, the composition of the asymmetric unit and the number and type of anomalous scatterers. However, the optimization of experimental parameters such as the wavelength used and the data quality (resolution limit, multiplicity and radiation damage) is critical for successful structure solution. Typically, the differences between the intensities of Friedel-related reflections are small: in SAD/MAD experiments the Bijvoet ratio Δ*F* ^±^/*F* is 2–6%, whereas in sulfur SAD (S-SAD) it can often be lower than 1% (Dauter, 2010 ▸). Single-wavelength anomalous diffraction (SAD) phasing has overtaken multiwavelength anomalous diffraction (MAD) in recent years (Rose & Wang, 2016 ▸). Since SAD data are generally collected from a single crystal, this also removes the problems of non-isomorphism that are commonly observed using the heavy-atom soaking methods single/multiple isomorphous replacement (SIR/MIR).

Atoms differ in the strength of their anomalous scattering, with heavy atoms such as Hg and Pt having a strong *f*′′ of ∼10 e at a wavelength below their *L*_III_ edges (Dauter *et al.*, 1999 ▸). Se provides a medium-strength anomalous signal (*f*′′ = 3.89 e) at its *K* edge, and selenium SAD is now the most common phasing method since the incorporation of selenomethionine into bacterially produced proteins is routine and also provides sequence-specific markers to guide the building process (Hendrickson *et al.*, 1990 ▸). The halide-soaking method pioneered by Dauter and coworkers has also been widely used and provides rapid derivatization of crystals via short cryo-soaks and the opportunity to collect and solve structures using the *f*′′(I) = 6.7 e anomalous signal on standard copper rotating-anode home sources in many cases (Abendroth *et al.*, 2011 ▸). The disadvantages of this method are that there is no predictable relationship between the number of sites and the content of the asymmetric unit, and many sites refine to low occupancy.

More recently, however, the trend has swung towards native SAD methods using the anomalous scattering signals of sulfur or phosphorus that are inherently present in the biological sample. This is in part owing to more challenging targets (large protein complexes, cell-surface receptors and membrane proteins) where protein expression is carried out in eukaryotic systems and selenomethionine labelling is problematic (Rose & Wang, 2016 ▸). These experiments not only rely on good crystals, but also on a good beamline setup and an optimized experiment to accurately measure weak anomalous signals at typical S-SAD data-collection energies (Cu *K*α, 8.18 keV, 1.5418 Å, 0.56 e or 6 keV, 2.066 Å, 0.95 e; Weiss, 2017 ▸). Higher flux beamlines, access to longer wavelengths and the option to use smaller crystals, which minimize absorption effects at longer wavelengths, are improving the success of S-SAD phasing. Recent S-SAD structures have highlighted that these experiments are not restricted to high-resolution cases (Akey *et al.*, 2014 ▸; Liu & Hendrickson, 2015 ▸); however, these cases have required the merging of many data sets from different crystals with careful scaling and selection of data sets, and using data with conservative resolution limits.

Experimental phasing clearly remains an important part of the structure-determination process and is often the only way to solve structures that comprise novel folds or for large flexible multiprotein complexes that often diffract to limited resolution and contain a high solvent content. We sought to make our approach complementary between new high-flux home sources such as the Bruker METALJET with a fixed wavelength of 1.34 Å (9.25 keV) and conventional synchrotron radiation. After first considering longer lanthanide-binding peptide sequences (Barthelmes *et al.*, 2011 ▸), we became aware of the naturally occurring copper-scavenging peptide GHK (glycine–histidine–lysine) and the related peptide DAHK (aspartate–alanine–histidine–lysine) (Hureau *et al.*, 2011 ▸). These peptides are found abundantly in human plasma, saliva and urine, and perform a scavenging role to tightly control the level of copper ions in the body (Pickart & Thaler, 1973 ▸; Pickart, 2008 ▸; Pickart *et al.*, 2012 ▸, 2017 ▸). The most important feature of the GHK peptide is its ability to form high-affinity complexes with copper(II) ions (Freedman *et al.*, 1982 ▸). The strength of copper-ion coordination to both GHK and its related peptide DAHK is high (7.0 ± 1.0 × 10^−14^ and 2.6 ± 0.4 × 10^−14^ *M*, respectively) as measured by ITC (Trapaidze *et al.*, 2012 ▸). This is weaker than commonly used chelators such as EDTA, and binding of the peptide to copper can be influenced both by its protonation state and by the presence of other potential chelators in the buffer (for example ammonium or Tris).

The GHK copper complex (GHK-Cu) has been extensively studied using many biophysical techniques, including X-ray crystallography as well as NMR and EPR spectroscopy (Hureau *et al.*, 2011 ▸; Freedman *et al.*, 1982 ▸; Laussac *et al.*, 1983 ▸). Structurally, the copper(II) ion is coordinated in a square-pyramidal coordination with the equatorial positions being occupied by three N atoms: the glycine amino-terminus, the amide N atom in the glycine–histidine peptide bond and the histidine side chain. The remaining equatorial and apical coordination positions are then completed in solution by labile ligands, allowing the formation of ternary species, such as with the carboxy-termini of symmetry-related molecules in the crystal structure (Hureau *et al.*, 2011 ▸). Similarly, we observed various symmetry-related amino-acid ligands being used to complete the coordination sphere in this work. We further show that an N-terminally fused GHK peptide can be utilized in standard crystallographic methods to enhance the crystallization of proteins and to provide reliable phasing sites for structure solution with minimal effort.

## 2. Materials and methods   

### 2.1. Protein expression and purification   

Genes were amplified by PCR using the oligonucleotides listed in Supplementary Table S1 and were cloned via Gibson assembly into a modified pQE80-based expression plasmid.

All proteins were expressed as N-terminal His14-bdSUMO-GHK fusions in *Escherichia coli* NEBExpress cells [*fhuA2 Δlon ompT gal sulA11 R(mcr-73::miniTn10–TetS)2 Δdcm R(zgb-210::Tn10–TetS) endA1 D(mcrCthe-mrr)114::IS10*]. Expression was carried out in TB medium, where cells were induced at an OD_600 nm_ of ∼0.8 with 150 µ*M* isopropyl β-d-1-thiogalactopyranoside and grown at 28°C for ∼4 h. Bacterial cells were resuspended in buffer consisting of 50 m*M* Tris–HCl pH 8.0, 500 m*M* NaCl, 20 m*M* imidazole pH 8.0, 2 m*M* dithiothreitol (DTT) and lysed by sonication on ice. Lysates were cleared by ultracentrifugation and the protein was bound in batch to a nickel(II) chelate matrix. The beads were then washed with buffer *A* supplemented with 30 m*M* imidazole, and the protein was finally eluted with 250 n*M* of the tag-cleaving bdSENP1 protease (Frey & Görlich, 2014*a* ▸,*b* ▸; Addgene pSF139 #104962). Before crystallization, the proteins were further purified on a Superdex 200 16/60 gel-filtration column (GE Healthcare) equilibrated with buffer consisting of 25 m*M* Tris–HCl pH 8.0, 250 m*M* NaCl, 2 m*M* DTT. The proteins were then concentrated to 9.5 mg ml^−1^ (342 µ*M*) GHK-GFP and 17 mg ml^−1^ (392 µ*M*) GHK-MBP-Nup98(1–29) before the addition of 1 m*M* copper(II) sulfate from a 100 m*M* stock solution. The proteins were then centrifuged to remove any precipitated material and frozen in liquid nitrogen before storage at −80°C. For GHK-Pfu polymerase 20 mg ml^−1^ (226 µ*M*) 0.5 m*M* copper(II) sulfate was added.

### 2.2. Crystallization   

For the crystallization of GHK-GFP, several initial crystallization conditions were identified in commercial grid and sparse-matrix screens using a Gryphon robot (Art Robbins) with 100 nl + 100 nl drops. The final crystallization conditions for crystal form I were 2 µl protein solution plus 2 µl reservoir solution consisting of 1.2–1.5 *M* ammonium sulfate, 0.1 *M* MES pH 6.5, 16% glycerol. Those for crystal form II were 2 µl protein solution plus 2 µl reservoir solution consisting of 20–30% MPD, sodium acetate pH 5.5, 0.1 *M* calcium chloride. Crystal form I was cryoprotected gradually by soaking in reservoir solution containing increasing amounts of glycerol up to 30% for several minutes before being mounted directly from the drop in a MiTeGen loop and flash-cooled in liquid nitrogen. Crystal form II was cooled in reservoir solution containing 30% MPD.

GHK-MBP-Nup98 crystallized from condition 1-29 of the Morpheus screen (Gorrec, 2009 ▸) using 2 µl protein solution plus 2 µl reservoir solution containing the NPS salt mix (0.09 *M* sodium nitrate, 0.09 *M* sodium phosphate, 0.09 *M* ammonium sulfate), 0.1 *M* buffer system 2 (sodium HEPES, MOPS pH 7.5) with precipitant mix 1 (24% PEG 500 MME, 12% PEG 20 000). As the crystals had already been grown under cryoprotectant conditions, they were cooled directly from the drop.

GHK-Pfu polymerase was crystallized using 2 µl protein solution plus 2 µl reservoir solution containing of 18% PEG 3350, 0.1 *M* Bis-Tris propane pH 5.5, 0.2 *M* ammonium sulfate, 50 m*M* manganese chloride, 8% glycerol. Crystals were cryoprotected gradually by soaking them in reservoir solution containing increasing amounts of glycerol up to 25%.

### 2.3. MALS   

Purified GHK-GFP (100 µl at 1 mg ml^−1^) was applied at 0.5 ml min^−1^ onto a Superdex 200 10/300 size-exclusion column (equilibrated in 50 m*M* Tris–HCl pH 7.5, 200 m*M* NaCl, 1 m*M* CuSO_4_). The column was coupled to a refractive-index (RI) detector (Shodex RI 101, Japan) and a multi-angle light-scattering (MALS) detector (Wyatt miniDAWN TREOS). The RI and MALS signals were used to detect the eluting complex and to deduce its absolute molecular mass using an algorithm implemented in the *ASTRA* 6.0 software package (Wyatt Technology). The complex runs as a single species on the gel-filtration column and showed no signs of aggregation or self-association. Multi-angle light scattering (MALS) detected a molecular mass of 27 kDa (the black line shows the fitting of the molecular mass), which matched the predicted mass of GHK-GFP with an error margin of ∼2.5%.

### 2.4. Crystallographic methods   

Diffraction data were collected at various wavelengths using a PILATUS 6M detector (Dectris, Baden, Switzerland) on the PXII beamline at the Swiss Light Source, Villigen, Switzerland or the P11 beamline at DESY, and all data were processed with *XDS* (Kabsch, 2010 ▸), *XSCALE* (Diederichs, 2006 ▸) and *POINTLESS*/*AIMLESS* (Evans & Murshudov, 2013 ▸; for statistics, see Table 1 ▸). In all cases initial phases were obtained by copper SAD (Cu-SAD) using the *CRANK*2 pipeline (Pannu *et al.*, 2011 ▸) with autobuilding using *Buccaneer* (Cowtan, 2012 ▸) and model building in *Coot* (Emsley *et al.*, 2010 ▸). Refinement was carried out using both *Phenix* (Liebschner *et al.*, 2010 ▸) and *REFMAC*5 (Kovalevskiy *et al.*, 2018 ▸), and refinement was validated using the *PDB-REDO* web server (Joosten *et al.*, 2014 ▸). For LLG completion, additional sites were added via the *Phaser* SAD routine. The average phase shift in degrees as a function of resolution was calculated using *SFTOOLS* (B. Hazes, unpublished work) (using the PHASHFT command) with respect to phases calculated for the final refined model.

### 2.5. Dose experiments   

Data sets were collected on EMBL beamline P14 at the PETRA III storage ring, DESY, Hamburg, Germany using a MD3 vertical-spindle diffractometer (EMBL and Arinax, Moirans, France) and an EIGER X 16M detector (Dectris). A collimated beam with a ‘top-hat’ beam profile was used for data collection. The beam dimensions (180 × 230 µm) were adjusted to the size of the crystal (180 × 180 × 230 µm). Diffraction data were collected using an incident flux of 1.6 × 10^12^ photons s^−1^ with 7.6 ms exposures, a φ-slicing of 0.10° and a total rotation range of 120° at 100% beam transmission, corresponding to a dose of 0.5 MGy. The same start oscillation angle and beamline settings for ten consecutive collected data sets were used to record a dose series for one crystal.

The diffraction data of the dose series were processed with *XDS* (Kabsch, 2010 ▸) and scaled with *XSCALE* (Diederichs, 2006 ▸). The anomalous substructure was determined by *SHELXD* (Schneider & Sheldrick, 2002 ▸). Phasing, density modification and model building were performed using either *CRANK*2 (Pannu *et al.*, 2011 ▸), including the protein sequence, or *SHELXE*, without prior phase information (Sheldrick, 2010 ▸). The radiation-induced damage was characterized using *RIDL* (Bury & Garman, 2018 ▸).

## 3. Results   

### 3.1. GHK protein production   

**Figure 1:**
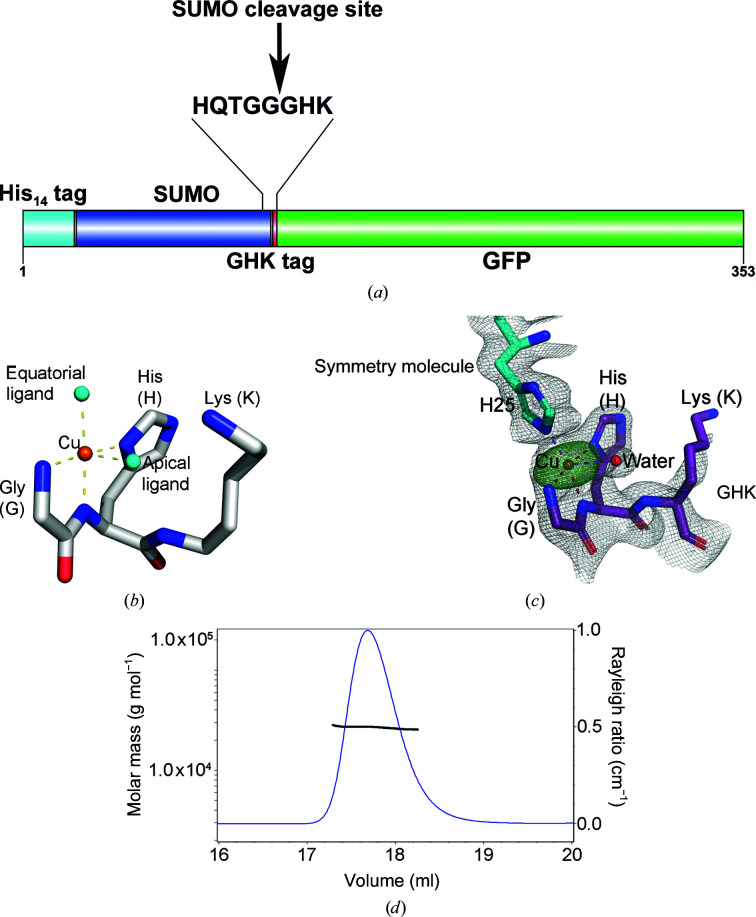
(*a*) Domain schematic for the production of GHK-tagged protein. (*b*) Representation of Cu-GHK coordination adapted from the coordinates of the Cu-GHK crystal structure CCDC-809108 {https://dx.doi.org/10.5517/ccw4y86; [copper(II)(GHK)]}. (*c*) The copper coordination in GHK-GFP crystal form I; the 2*F*_o_ − *F*_c_ map is contoured at 1σ and the *REFMAC*5 anomalous difference map is contoured at 10σ. (*d*) MALS analysis of GHK-GFP performed in the presence of 1 m*M* CuSO_4_, showing a molecular weight of 2.700 × 10^4^ kDa (±0.428%); the theoretical monomer molecular weight is 2.631 × 10^4^ kDa.

The GHK peptide tag is a small rigid motif that should provide excellent anomalous scattering from its bound copper(II) ion at 8.9 keV (Fig. 1 ▸ *a*) and should therefore be very useful in experimental phasing. When generating GHK fusion proteins, a free amino-terminal glycine is required since it forms a critical part of the square-pyramidal copper coordination (Fig. 1 ▸ *b*). To generate such an N-terminus, our constructs contained a His_14_-SUMO tag followed by the GHK-tag sequence appended to the N-terminus of the protein of interest. The subsequent cleavage of the His_14_-SUMO tag with the highly active tag-cleaving bdSENP1 protease (Frey & Görlich, 2014*a* ▸,*b* ▸) yields GHK-tagged protein. Other proteases commonly used in protein preparation for structural studies might also be used in a similar manner depending upon their recognition sequence, *i.e.* provided that they allow a glycine in the P1′ position and a histidine in the P2′ position (for example an ENLYFQ/G TEV cleavage site should also work as well). The high-affinity binding of copper(II) to the GHK sequence means that only equimolar stoichiometries are required for full occupancy and that the bound copper(II) resists reducing agents such as DTT (Freedman *et al.*, 1982 ▸); however, in these experiments we used a 2–3 molar excess of copper(II) sulfate.

#### 3.1.1. SAD phasing of an sffrGFP6 variant using a GHK-Cu tag   

As a proof of principle, we first tested the GHK tag on crystals of sffrGFP6, which is a surface-modified GFP variant that passes nuclear pore complexes 100-fold faster than standard eGFP (Frey *et al.*, 2018 ▸). This variant could be expressed in large amounts and showed good crystallization potential when fused to a series of other short peptide sequences (Huyton & Görlich, unpublished work). Multi-angle light scattering (MALS) coupled to gel-filtration analysis confirmed that GHK-GFP behaved as a monomer in solution and that this was unchanged in the presence of 1 m*M* copper(II) ions (Fig. 1 ▸ *d*). The engineered Cu-GHK-GFP fusion protein crystallized readily using diverse precipitants including ammonium sulfate and 2-methyl-2,4-pentanediol (MPD), and data were collected from these two crystal forms.

X-ray diffraction data were initially collected from crystals using an in-house METALJET X-ray source (Bruker) and then using synchrotron radiation at a wavelength of 1.34 Å (9.25 keV), which proved excellent for Cu-SAD experiments (data-collection statistics are shown in Table 1 ▸). At a wavelength of 1.34 Å the anomalous scattering for copper is 3.69 e (Supplementary Fig. S1*a*; http://skuld.bmsc.washington.edu/scatter/) and is similar to the scattering of selenium at its *K* edge (3.84 e; Supplementary Fig. S1*a*). The calculated Bijvoet ratio for both crystal forms with two molecules per asymmetric unit (2 × 26.3 kDa) and two bound copper ions is 1.72% (https://bl831.als.lbl.gov/xtalsize.html).

#### 3.1.2. GHK-GFP crystal form I   

The phasing and structure determination proved to be straightforward using the *CRANK*2 pipeline (Pannu *et al.*, 2011 ▸; Fig. 1 ▸ *c*, Supplementary Figs. S2*a* and S2*b*). In fact, for crystal form I in space group *P*6_5_22 the structure could be easily solved using a minimal complete data set for this space group with only 30° of data (Supplementary Figs. S2*c* and S2*d*), which prompted us to further investigate the phasing limits of single GHK-Cu sites. This structure was refined to 1.68 Å resolution and shows a square-pyramidal coordination of copper(II) ions: in the equatorial plane by the glycine amino N atom, the amide N atom of the glycine–histidine peptide bond and the N atom of the histidine side chain. The additional equatorial and apical ligands in this case are provided by the side chain of His25 from a symmetry-related molecule and an ordered water molecule (Fig. 1 ▸ *c*).

#### 3.1.3. Increased phasing power by combined Cu-SAD and S-SAD phasing   

**Figure 2:**
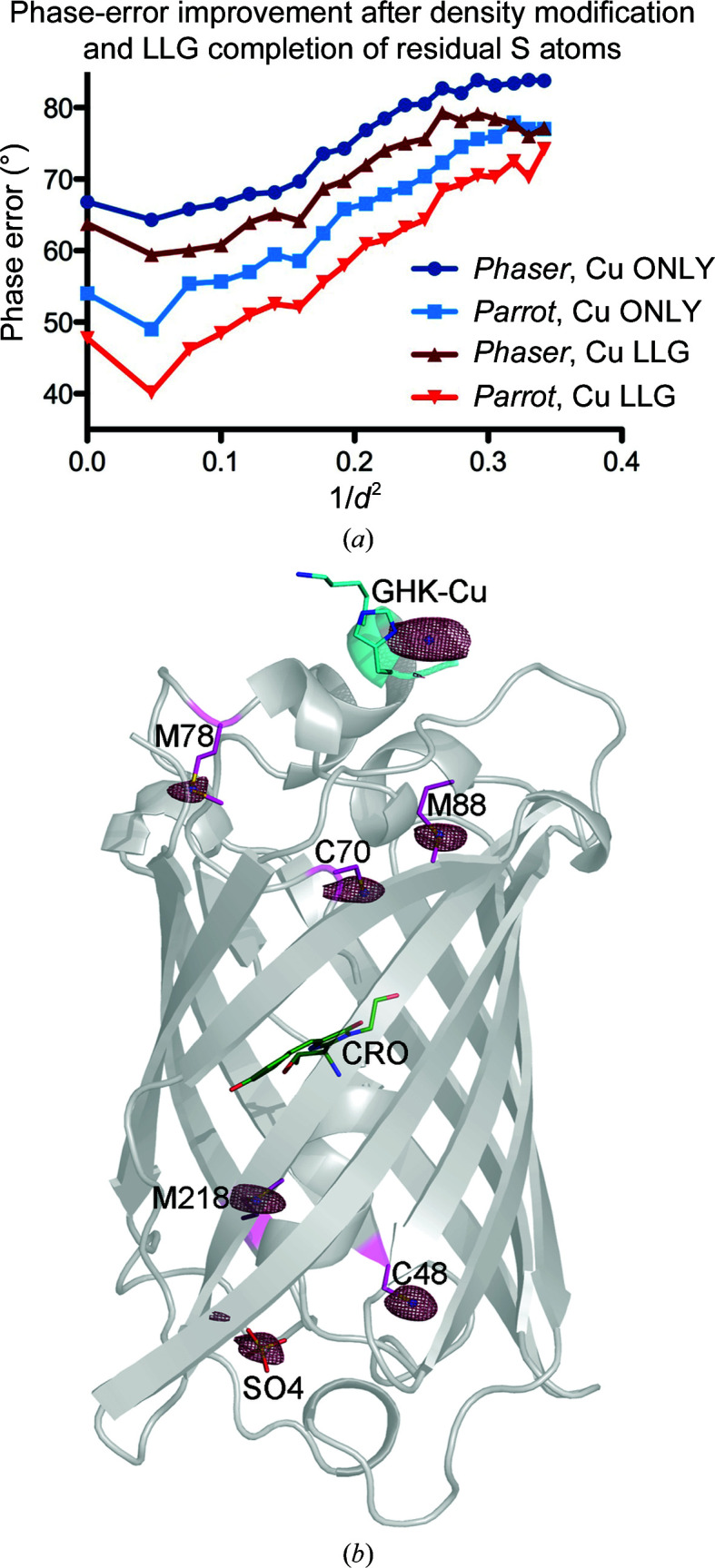
(*a*) A comparison of the improvements in phase error as calculated with *SFTOOLS* before and after the inclusion of LLG map completion with residual sulfur sites and with density modification using *Parrot*. (*b*) The structure of GHK-GFP crystal form I is shown as a cartoon in grey. The GHK motif is highlighted in cyan. Native sulfur-containing residues are shown as sticks, with *Phaser*-refined S atoms from LLG maps shown as blue spheres. The *REFMAC*5 anomalous difference map is contoured at 4σ. For clarity only one chain is shown.

At a wavelength of 1.34 Å (9.25 keV) the anomalous scattering from native S atoms is 0.428 e, which is much weaker than the more typical S-SAD data-collection energies (Cu *K*α, 8.18 keV, 1.5418 Å, 0.56 e or 6 keV, 2.066 Å, 0.95 e; Weiss, 2017 ▸). The log-likelihood gradient (LLG) map shows the difference in anomalous scattering between the current substructure and the true contents of the crystal. Despite being able to automatically build the GHK-Cu-GFP structure solely from the copper(II) site phases, we compared the mean phase error improvement after LLG completion of anomalous scatterers with S atoms using the *Phaser* SAD pipeline (Read & McCoy, 2011 ▸). The differences in the mean phase error against resolution for refined Cu-atom sites, as well as sulfur LLG completion, before and after density modification with *Parrot*, are shown in Fig. 2 ▸(*a*) and resulted in an average phase-quality improvement of 5°. The LLG completion-refined sites contain the positions of ten S atoms and one highly ordered sulfate ion, as depicted by the anomalous difference map of the fully refined structure (Fig. 2 ▸ *b*). Here, the peak heights for the bound Cu atoms are 50σ and 70σ, respectively, while the heights of protein S atoms and ordered sulfates are in the range 5.6–9.4σ. Although small, a noticeable difference in map quality was observed from the phase-quality improvement of 5°, as illustrated in Supplementary Fig. S3.

#### 3.1.4. Cu-SAD data collection by titrating anomalous signal at 17 keV for GHK-GFP crystal form I   

To examine the phasing limit of the GHK tag, we collected highly redundant Cu-SAD data at a very short wavelength (17 keV, 0.73 Å), where the anomalous scattering from copper is 1.33 e and the calculated Bijvoet ratio for GHK-GFP of 0.62% is significantly smaller and close to the theoretical limit for structure solution (Wang, 1985 ▸). We collected a total of 4 × 360° sweeps (1440°) from four areas of the same crystal (see Table 1 ▸). A successful structure solution with almost complete autobuilding could be achieved with 2–4 sweeps using the *CRANK*2 pipeline (Pannu *et al.*, 2011 ▸; Table 1 ▸; Supplementary Figs. S2*e* and S2*f*).

#### 3.1.5. Dose-dependent effects on the phasing power of GHK-GFP crystal form I   

The X-ray dose that a cryocooled crystal can absorb before the diffraction pattern decays to half of its original intensity is termed the Henderson limit and has been calculated to be 20 MGy (Henderson, 1990 ▸). Radiation damage clearly occurs much earlier than the Henderson limit and is an important parameter to consider in experimental phasing. The phasing power of heavy atoms deteriorates in a dose-dependent manner, and loss of phasing power can be attributed to increased global disorder during the experiment that is caused by cell expansion and structural changes/movement of molecules within the unit cell. For bound metal ions, specific radiation damage around their coordination centres often occurs at a much lower dose (Holton, 2009 ▸). Radiation damage can often lead to failure in solving structures by MAD/SAD techniques owing to deterioration of the small signal, which is typically less than 2–6%. For selenomethionine-substituted crystals typical doses of 2–5 MGy are common for structure solution, with the lowest life dose being measured as 2 MGy by X-ray absorbance near-edge spectrum (XANES) experiments (Holton, 2009 ▸). In contrast, the radiation-sensitivity of some metalloproteins can be as low as 0.3 MGy (Corbett *et al.*, 2007 ▸).

To ascertain the radiation-sensitivity of the GHK-Cu tag, we collected a series of data sets with increasing dose on EMBL beamline P14 at the PETRA III storage ring at DESY, Hamburg, Germany. Here, data collection using a CRL collimated X-ray beam with a ‘top-hat’ profile allows optimal exposure of the whole crystal and more accurate estimation of the applied dose (Schrader *et al.*, 2016 ▸). A total of 10 × 0.5 MGy data sets were collected both at the copper absorption edge of 8.9994 keV (4.44 e) and at a remote energy of 12.7 keV (2.2 e) (http://skuld.bmsc.washington.edu/scatter/); the fluorescence scan is shown in Supplementary Fig. S4. The global influence of radiation damage as a function of resolution was evident as a decrease in both the high-resolution diffraction limit (cutoff of *I*/σ = 1.0) and anomalous signal (cutoff CC_1/2,anom_ = 30%) (Supplementary Fig. S5). In both data sets the structures could easily be solved using *CRANK*2, even using radiation-damaged data with a 5 MGy dose. Structure solution was also possible with *SHELXC*/*D*/*E* (Sheldrick, 2010 ▸), as implemented in *HKL*2*MAP* (Pape & Schneider, 2004 ▸). The anomalous substructures of the early (0.5 MGy) and radiation-damaged (5 MGy) data sets could be solved by *SHELXD* (Supplementary Fig. S6*a*). For the 0.5 MGy data sets measured both at the anomalous scattering edge of 8.999 keV and the remote energy of 12.7 keV, 399 (86%) and 366 (79%) residues were built by *SHELXE* within ten cycles of autotracing. In contrast, in the respective 5 MGy data sets 193 (42%) and 125 (27%) residues were autotraced by *SHELXE* (Supplementary Fig. S6*c*). This highlights the importance of using model-based phases implemented in the autotracing module of *CRANK*2 for *de novo* phasing of copper(II)-GHK-GFP and that this becomes increasingly dominant as the radiation dose increases.

**Figure 3:**
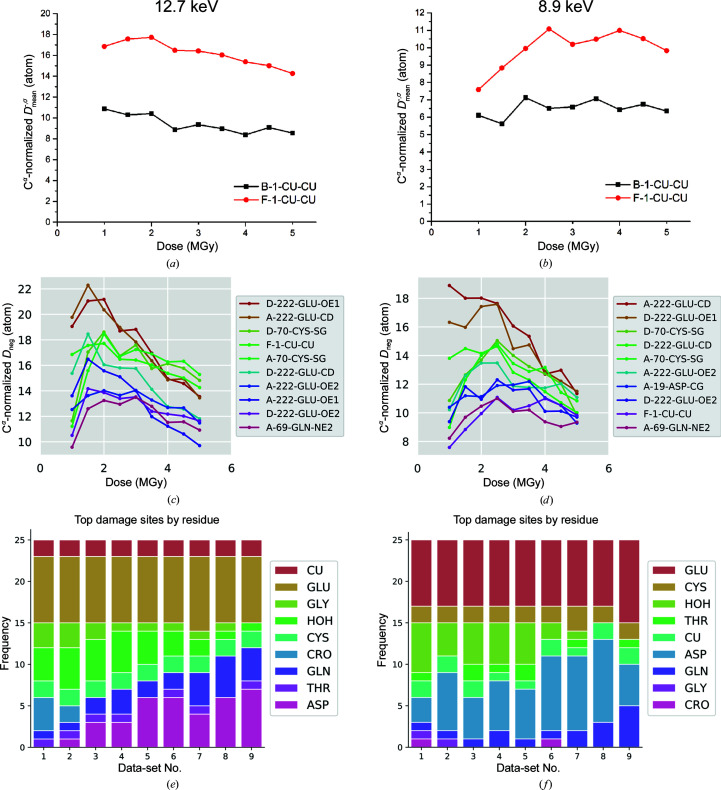
Radiation-damage analysis of the GFP-GHK dose series performed with *RIDL*. (*a*, *b*) The plot represents the *D*_neg_ values of the two Cu atoms in the asymmetric unit normalized against the C^α^ atoms. The *D*_neg_ values, which describe the extent of radiation damage, are plotted for each data set of the dose series measured at (*a*) 12.7 keV and (*b*) 8.99 keV. The *D*_neg_ values of the top ten damaged atoms acquired at 12.7 and 8.99 keV are shown in (*c*) and (*d*), respectively. The column graphs in (*e*) and (*f*) depict the residue-type distribution of the top 25 radiation-damaged sites of the dose series acquired at 12.7 and 8.99 keV, respectively.

Cys S^γ^, Asp O^δ^ and Glu O^∊^ atoms in protein crystals are known to be susceptible to specific radiation damage (Gerstel *et al.*, 2015 ▸). The radiation-induced damage was assessed using *RIDL* (Bury & Garman, 2018 ▸). Specific damage can be visualized by the calculation of Fourier difference density maps and comparison of the per-atom metric of electron-density changes (C^α^-normalized *D*_neg_ values, calculated by *RIDL*), which have been proposed to be the most meaningful way to compare independently conducted damage experiments (Bury & Garman, 2018 ▸). In our experiments, the two GHK-coordinated copper ions in the asymmetric unit (F-1-C and B-1-Cu) are not equally sensitive to radiation damage (Figs. 3 ▸ *a*, 3 ▸ *b* and Supplementary Fig. S7). The slightly lower occupancy of F-Cu possibly suggests a weaker coordination of the GHK tripeptide, since the copper-coordinating glycine residue for this site is also found in the top 25 damage sites (Figs. 3 ▸ *e* and 3 ▸ *f*). For both the 12.7 and 8.9 keV data sets, the top ten radiation-damage atom list obtained from the *RIDL* analysis contains atoms belonging to known radiation-sensitive atoms as well as one of the GHK-bound copper ions (Figs. 3 ▸ *c* and 3 ▸ *d*). The residues Glu222, Cys70, Asp19 and Gln69 all surround the GFP active-site chromophore and this is the main region of local radiation damage. Indeed, the decarboxylation of Glu222 has been observed in several other studies with a low dose of 0.5 MGy. Exposure at low dose first leads to a rearrangement in the crystals as indicated by the higher radiation-damage values. After an initial increase in the occupancy of the relevant atoms, the occupancy values decrease as a consequence of radiation damage (Figs. 3 ▸ *c* and 3 ▸ *d*). These data suggest that the radiation-sensitivity of the GHK-bound copper ions is in the same range as observed for known radiation-sensitive residues and that this is around 2–3 MGy (Figs. 3 ▸ *c* and 3 ▸ *d*). This might explain the robustness of the Cu-GHK phasing procedure compared with other heavy atoms such as the divalent *d*-block metal cations such as Zn^2+^, Mn^2+^ and Fe^2+^ and alternative Cu^2+^ coordinations, which are described to exhibit stronger radiation-sensitivity and appear to be less reliable for the success of phasing (Corbett *et al.*, 2007 ▸; Meyer *et al.*, 2006 ▸; Yano *et al.*, 2005 ▸). This has been shown to be as little as 0.3 MGy for the metalloprotein putidaredoxin (Corbett *et al.*, 2007 ▸).

The local radiation damage of the 8.99 keV dose series (Fig. 3 ▸ *d*) is smaller than the local radiation damage of the 12.7 keV dose series (Fig. 3 ▸ *c*). This is a result of the higher global radiation damage at 8.99 keV (Supplementary Fig. S5). The same phenomenon causes the decrease in the C^α^-normalized *D*_neg_ values throughout the dose series (Figs. 3 ▸ *c* and 3 ▸ *d*). In summary, for the measured dose range, radiation damage does not significantly compromise the use of copper for phasing. The rigidity of Cu-GHK recognition and its high occupancy is correlated with the robustness of phasing and appears to be the reason for its success at low Bijvoet ratios.

### 3.2. Crystal form II of GHK-GFP   

**Figure 4:**
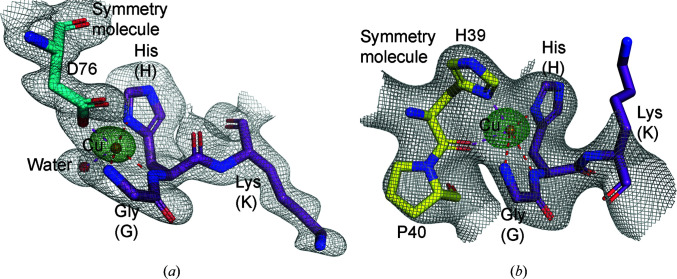
The copper coordination in the (*a*) GHK-GFP crystal form II and (*b*) GHK-MBP-Nup98 structures. The 2*F*_o_ − *F*_c_ map is contoured at 1σ and the *REFMAC*5 anomalous difference map is contoured at 10σ.

GFP proteins have commonly been crystallized in MPD with Ca^2+^ ions, and a second crystal form of GHK-GFP could be grown at pH 5.5 under these conditions. High-resolution data (1 × 360°) were collected at a wavelength of 1.34 Å from thin plate-like crystals to 1.3 Å resolution. Structure solution was again trivial using the *CRANK*2 pipeline (Pannu *et al.*, 2011 ▸) and almost an entire model could be built automatically (Table 1 ▸, Fig. 4 ▸ *a*, Supplementary Figs. S8*a* and S8*b*). The GHK tag in crystal form II shows completion of the coordination of copper(II) ions in the equatorial plane by the side chain of Asp76 from a symmetry-related molecule, since at this pH the other histidine side chains will be protonated. The apical coordination was again provided via an ordered water molecule (Fig. 4 ▸ *a*). In this crystal form, two additional copper sites were identified, one in each molecule in the asymmetic unit. The first is a weakly ordered site with an occupancy of 0.29 coordinated by chain *A* residues His25 and Glu132. The second was a highly ordered site occupancy of 1.0 coordinated by chain *C* residues His25 and Glu132 but with additional coordination by His77 from a symmetry-related molecule. Calcium ions were also present in the crystallization conditions and we modelled an octahedral calcium site in each molecule in the asymmetic unit, with occupancies of 0.56 and 0.48, that are coordinated by Glu142 and water molecules. The sites were verified using the *CheckMyMetal* (*CMM*) server (Zheng *et al.*, 2017 ▸).

### 3.3. Cu-SAD data collection and phasing of GHK-MBP-Nup98   

To test our predictions that single-site GHK-Cu phasing could be universally applicable, we cloned, expressed and crystallized a complex between maltose-binding protein and a 29-residue FG-containing peptide from the amino-terminal region of *Tetrahymena thermophila* MacNup98a (Iwamoto *et al.*, 2009 ▸; Schmidt & Görlich, 2015 ▸). The non-GHK-tagged protein had previously undergone screening using 14 commercial screens and failed to produce any hits; however, the GHK-tagged protein produced six hits in these commercial crystallization screens, with condition 1-29 from the Morpheus screen (Gorrec, 2009 ▸) producing the best crystals. From this crystal form, we collected 3 × 360° (1080°) of data at different positions within the same crystal. The data were collected at 1.3776 Å (9.0 keV) with an anomalous scattering of 3.88 e for Cu. The theoretical Bijvoet ratio for GHK-MBP-Nup98 (12 × 43.3 kDa) at this wavelength was 1.41%. The diffraction extended to 2.7 Å resolution and, although the anomalous signal was much weaker than for the GHK-GFP cases (Table 1 ▸, Supplementary Figs. S8*c* and S8*d*), automatic structure solution was again possible using *CRANK*2 (Supplementary Figs. S8*c* and S8*d*). The final structure contained 12 molecules per asymmetric unit. All copper sites refined with an occupancy of 1.0 but with an unusual copper(II) coordination. The equatorial plane ligand and the apical water molecule are replaced by residues His39-Pro40 from a symmetry molecule. The histidine side chain again acts as the equatorial plane ligand and the main-chain carbonyl O atom of the histidine completes the coordination in place of an apical water molecule (Fig. 4 ▸ *b*). Unfortunately, the C-terminal FG-containing peptide is highly disordered in the crystal structure and we were unable to visualize the interactions between FG motifs (Supplementary Figs. S9*a* and S9*b*). That the GHK-MBP construct nevertheless showed such a good crystallization propensity is promising and demonstrates that the GHK tag could be an excellent choice when fused to other difficult-to-crystallize targets.

## 4. Discussion   

The concept for the GHK method arose after searching for atoms with absorption edges close to the 1.34 Å wavelength of our newly installed METALJET home source (Bruker) and reading the excellent work of Yeates and coworkers (Laganowsky *et al.*, 2011 ▸), where specific metal-ion sites were engineered into helices of well characterized proteins in order to enhance symmetry in crystallization. Our approach requires little effort to engineer the GHK tag onto the protein of interest and has the advantage that it requires no previous structural knowledge. The GHK tag has limitations as it is restricted to just one tag at the N-terminus of a protein and is limited to the use of buffers that do not contain chelators such as imidazole or EDTA. However, the GHK tag allows a new area of crystallization space to be explored since the equatorial plane and apical coordination are not fixed to one particular amino acid. The GHK tag is also small enough to have minimal effects on ‘native’ protein crystallization and can therefore be screened in parallel both with and without copper(II) ions in a ‘two proteins for one’ approach. Although we chose the natural tripeptide GHK here, the dipeptide GH essentially behaves in the same way as the lysine side chain is not involved in copper coordination (Blount *et al.*, 1967 ▸).

The peptides GHK and DAHK are natural copper-binding sequences and both the DAHK and DAH sequences have been shown to bind the metal ions nickel, cobalt and copper (Predki *et al.*, 1992 ▸). The use of metal ions other than Cu might be beneficial for some purposes. While these elements all have a similar anomalous signal at their respective absorption edges (Ni *K* edge, 8.3 keV, 1.48 Å, *f*′′ = 3.9 e; Co *K* edge, 7.7 keV, 1.60 Å, *f*′′ = 3.9 e; Cu *K* edge, 8.97 keV, 1.38 Å, *f*′′ = 3.9 e), it may be prudent to use the longer wavelength *K* edges of Ni and Co to maximize the anomalous signal of residual S atoms. A comprehensive study of copper-binding tripeptide sequences has been carried out (Khoury *et al.*, 2014 ▸) and we are currently working to test these sequence variations to customize or improve the tag. The evidence suggests that the position of histidine within a tripeptide promotes a different coordination. While GHE should coordinate similarly to the natural GHK peptide, other sequences such as EGH or HEG should form an internal copper-binding scaffold, removing the crystallization-promoting effects of GHK. More interestingly, the sequence variation HH(E/G) leads to the formation of a 2:2 dimer (Khoury *et al.*, 2014 ▸), allowing an alternative screening of crystallization space.

In this work, we have shown that the GHK tag could be used to solve two crystal forms of the sffrGFP6 variant and an MBP fusion protein using the *CRANK*2 phasing pipeline. One disadvantage that we have observed is the tendency to produce larger unit-cell dimensions in some cases. As part of this work, we also collected data from crystals of GHK-Pfu polymerase (data not shown). In this case, the small ∼30 µm cube-shaped crystals with large unit-cell dimensions (*P*6_5_22, *a* = *b* = 79, *c* = 550 Å, α = β = 90.0, γ = 120.0°) allowed data collection to only 4.0–4.5 Å resolution. Although sites could be found using *SHELXD* (Supplementary Figs. S6*e* and S6*f*), the signal was insufficient for density modification to break the phase ambiguity and produce an interpretable map.

The elements copper and zinc are neighbours in the periodic table and they show very similar anomalous scattering properties at their respective absorption edges (http://skuld.bmsc.washington.edu/scatter/; Cu *K* edge, 1.380 Å, 3.90 e; Zn *K* edge, 1.283 Å, 3.89 e). An excellent example showing the phasing limits of such elements is the phasing of RNA polymerase II that was achieved by multicrystal zinc MAD phasing at 4.1 Å resolution with 570 amino acids per Zn atom (Meyer *et al.*, 2006 ▸). Additional computational simulation of these MAD data suggested a phasing limitation of 1100 amino acids per Zn atom (Meyer *et al.*, 2006 ▸). In this comparison the Zn sites were also identified using anomalous difference Fourier maps calculated with model-derived phases, highlighting that MR-MAD or MR-SAD may be more applicable in some cases where resolution is limiting.

Radiation damage significantly increases above a wavelength of 1.3 Å (Müller *et al.*, 2002 ▸) and optimal data collection is a compromise between dose, signal absorption and radiation damage (Weiss, 2017 ▸). Here, we have shown that GHK-Cu is rather resistant to damage and could be solved using a 5 MGy data set at its absorption edge at 8.9 keV. Our GHK Cu-SAD technique is complementary to the use of SAD and MR-SAD phasing methods that are being developed further to solve the structures of challenging projects, especially where selenium incorporation poses problems and only native crystals can be obtained (Read & McCoy, 2011 ▸; Skubák *et al.*, 2018 ▸; Rose *et al.*, 2015 ▸). The application of a GHK tag in the *de novo* phasing of XFEL data may also prove to be beneficial (Nass *et al.*, 2016 ▸; Schlichting, 2017 ▸).

We anticipate that the GHK tag may be beneficial to other crystallographic problems such as membrane-protein crystallization, where it is conceivable that an N-terminal GHK tag and histidine-scanning point mutations in loop regions might serve to expand crystallization space without the need to develop antibodies or nanobodies as crystallization chaperones to provide crystal contacts. Alternatively, when such crystallization chaperones already exist, one can also expand them further to be useful phasing reagents by the simple addition of a GHK tag.

Although the metal-binding and crystallization-promoting activities of these sequence tags are critical to their use, it must be clear that minimizing any inherent flexibility of the protein termini will increase the chance of success, and accurate domain boundaries should be determined from homology modelling or protease digest experiments. The GHK tag will be a useful addition to the crystallographer’s ‘phasing toolbox’ and we hope to develop it further and demonstrate its use in more examples in the near future.

## Supplementary Material

PDB reference: GHK-MBP-Nup98, 6qug

PDB reference: GHK-GFP, form 1, 6quh

PDB reference: GHK-GFP, form 1, 17 keV, 6qui

PDB reference: GHK-GFP, form 2, 6quj

Supplementary Figures and Table. DOI: 10.1107/S2059798320013741/nj5295sup1.pdf

## Figures and Tables

**Table 1:** Data-collection and refinement statistics

Structure	GHK-GFP, form 1	GHK-GFP, form 1, 17 keV	GHK-GFP, form 2	GHK-MBP-Nip98(1–29)
PDB code	6quj	6qui	6quh	6qug
Data collection				
No. of crystals	1	1	1	1
Data (°)	1360	1440	1360	1040
Wavelength (Å)	1.399	0.766	1.340	1.376
Space group	*P*6_5_22	*P*6_5_22	*P*22_1_2_1_	*P*12_1_1
*a*, *b*, *c* (Å)	85.9, 85.9, 270.1	86.2, 86.2, 269.5	53.4, 99.3, 102.0	82.9, 286.7, 110.7
α, β, γ (°)	90, 90, 120	90, 90, 120	90, 90, 90	90, 92, 90
Resolution (Å)	50.0–1.68	44.9–1.93	71.1–1.50	47.9–2.70
Completeness (%)	99.6 (99.6)	99.8 (99.5)	99.6 (99.1)	99.9 (99.9)
*R*_merge_	0.059 (2.36)	0.151 (2.79)	0.150 (1.75)	0.210 (1.59)
*R*_p.i.m._	0.009 (0.49)	0.012 (0.22)	0.043 (0.52)	0.048 (0.41)
Mean *I*/σ(*I*)	37.69 (1.19)	40.11 (2.19)	22.72 (0.74)	11.74 (1.92)
No. of unique reflections	68342	45000	87645	141017
Multiplicity	36.2 (23.1)	155.8 (148)	12.3 (12.0)	19.8 (15.6)
CC_1/2_	1 (0.63)	1 (0.91)	0.99 (0.46)	0.99 (0.65)
*CRANK*2				
No. of Cu sites	2	2	2	12
Residues built	468	435	462	4354
*R*_work_/*R*_free_ (%)	35.9/40.3	37.5/42.6	24.2/26.7	27.6/32.4
Refinement				
Resolution (Å)	50.0–1.68	44.9–1.93	71.1–1.5	47.9–2.7
No. of reflections	64887	42789	83308	133953
*R*_work_ (%)	18.04 (20.03)	20.80 (26.40)	14.20 (27.46)	20.73 (30.57)
*R*_free_ (%)	22.02 (27.19)	22.98 (31.23)	18.75 (32.52)	22.64 (33.06)
No. of atoms				
Protein	3688	3677	3739	34298
Ligand/ion	73	73	98	323
Water	269	259	588	272
Wilson *B* value (Å^2^)	24.25	35.63	18.52	57.96
Mean *B* values (Å^2^)				
Protein	43.04	39.95	21.45	66.65
Ligand/ion	44.33	40.49	28.59	53.56
Water	53.61	44.53	40.27	49.33
R.m.s. deviations				
Bond lengths (Å)	0.006	0.005	0.007	0.007
Bond angles (°)	1.330	1.314	1.407	1.339
Ramachandran analysis (%)				
Preferred	98.11	99.11	98.88	98.56
Allowed	0.89	0.89	1.12	1.44
Outliers	0	0	0	0
Clashscore	3.8	2.1	2.2	3.3
