# Supplementary material for: The copper(II)-binding tripeptide GHK, a valuable crystallization and phasing tag for macromolecular crystallography
Source: Acta Crystallogr D Struct Biol. 2020 Nov 19;76(Pt 12):1222–32. doi: 10.1107/S2059798320013741 (PMC7709198; doi:10.1107/S2059798320013741)
Supplement: Supplementary file 1 [file d-76-01222-sup1.pdf]

STRUCTURAL
BIOLOGY

Volume 76 (2020)

Supporting information for article:

The copper(II)-binding tripeptide GHK, a valuable crystallization and phasing tag for macromolecular crystallography

Alexander Mehr, Fabian Henneberg, Ashwin Chari, Dirk Görlich and Trevor Huyton

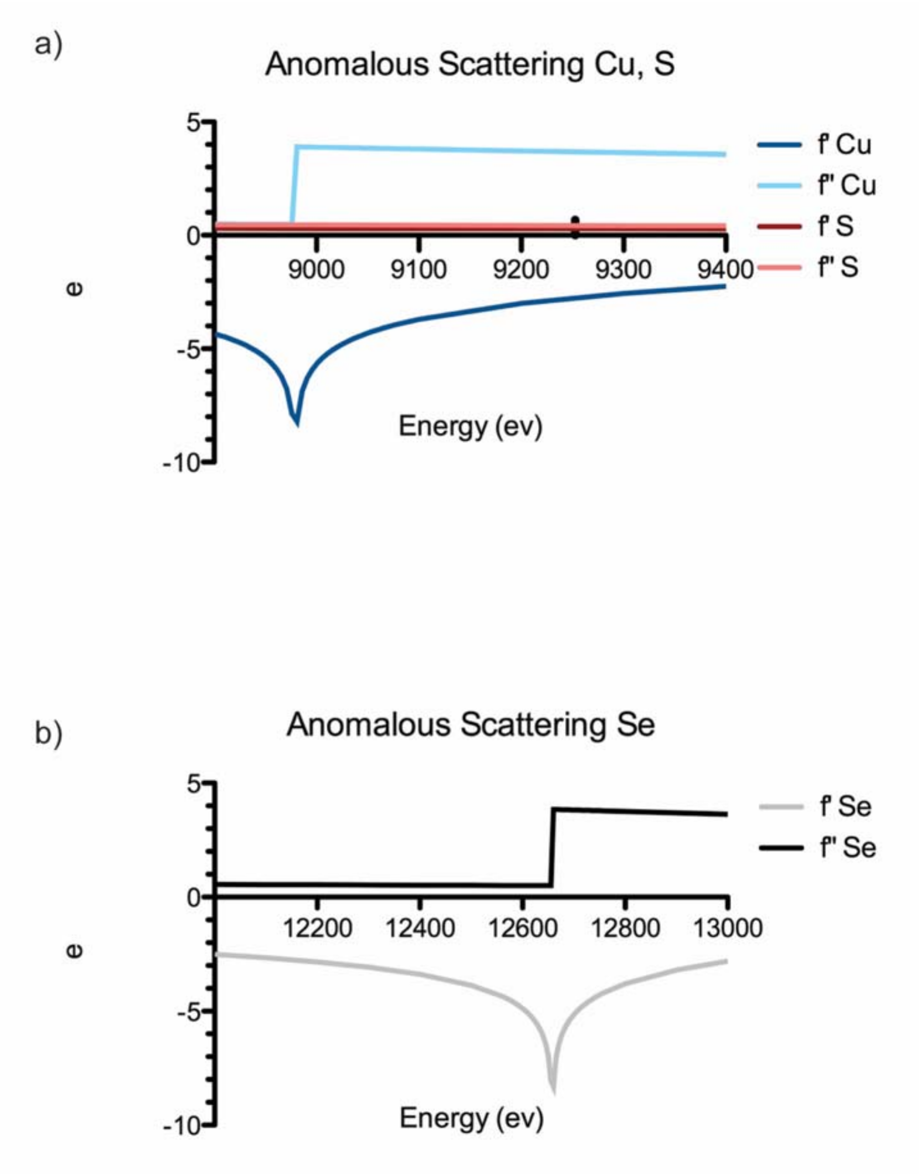

Figure S1 a) The theoretical anomalous scattering for Cu and S (between 8900 and 9400 eV) is 3.69 é at 9252.5 eV (1.34 Å) b) Comparison with the scattering of 3.84 é for selenium at its K-edge 12660.0 eV (0.9793 Å). Data points were plotted from the webserver http://skuld.bmsc.washington.edu/scatter/

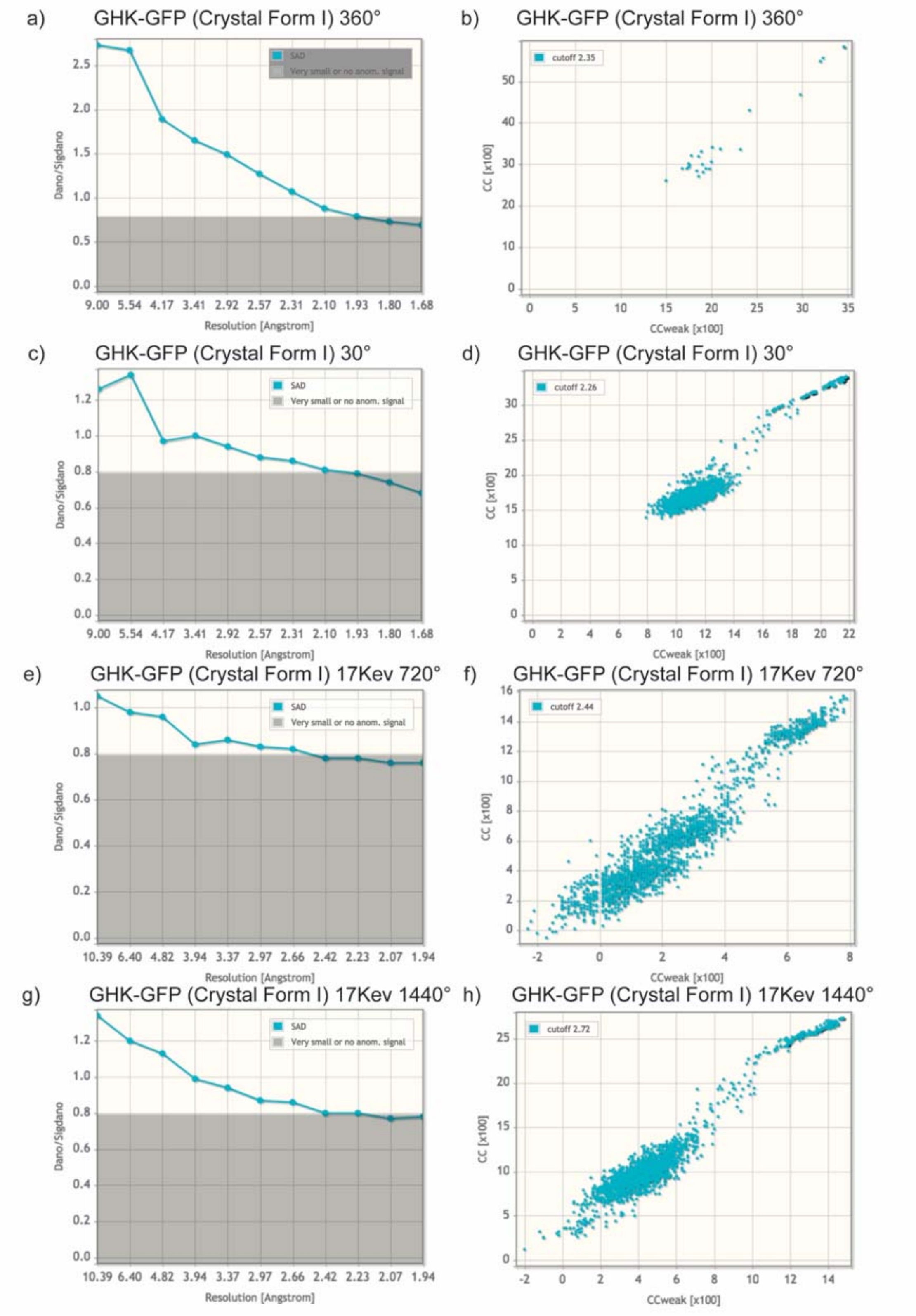

Figure S2 Output from *CRANK2* pipeline run via *CCP*4 online (https://ccp4serv7.rc-harwell.ac.uk/ccp4online) for GHK-GFP crystal form I. Panels a,c,e,g; Anomalous signal estimation output from *SHELXC*. Panels b,d,f,h; Profiles of *SHELXD* correlation coefficients (CC) for substructure determination. Results are shown for the datasets given in Table 1.

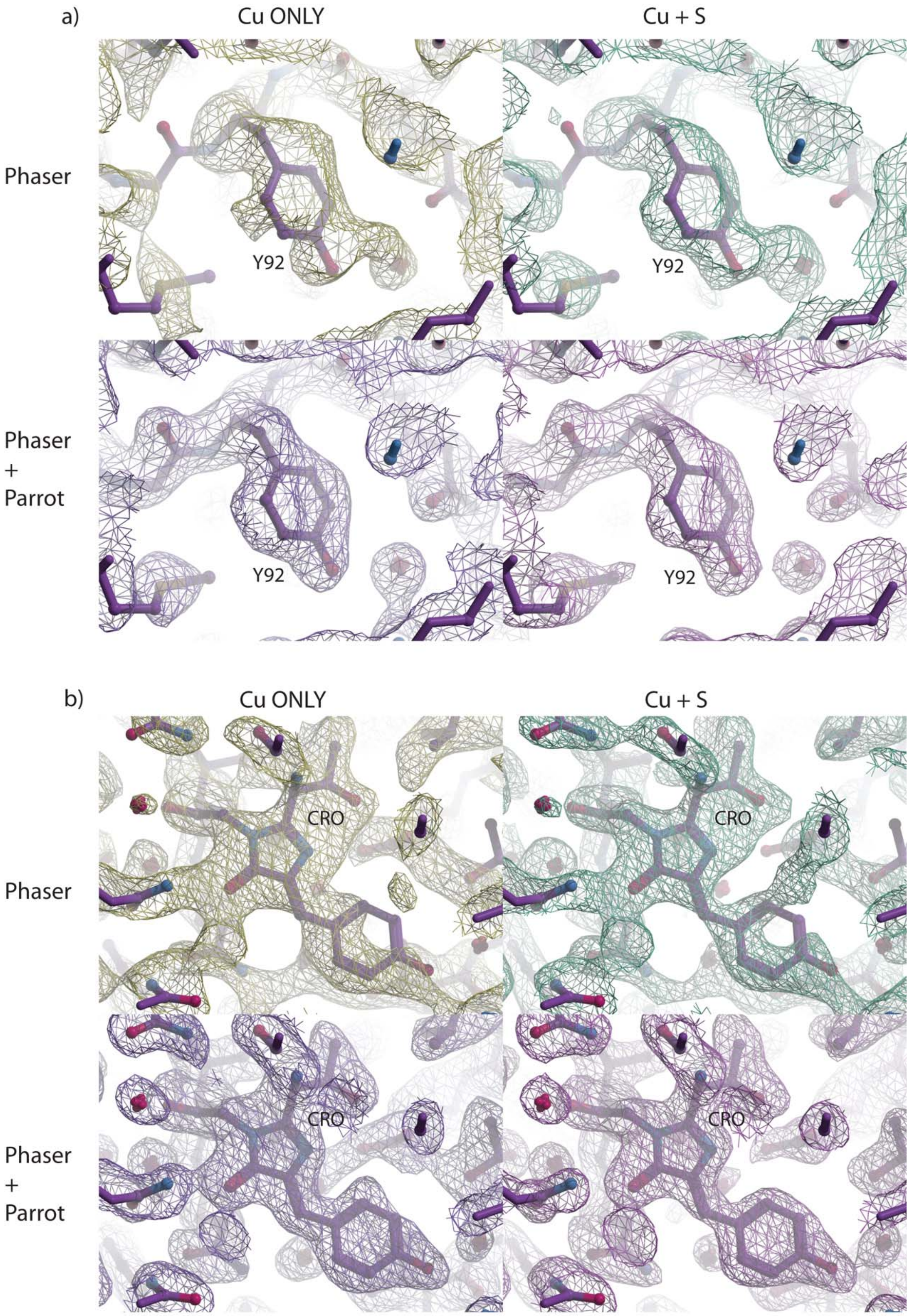

Figure S3 The effects of phase error improvement after LLG completion of residual sulfur atoms, maps without density modification (*Phaser*) or after density modification (*Phaser* + *parrot*) are contoured at 1σ a) Surrounding residue Y^{92}, b) surrounding the GFP chromophore CRO.

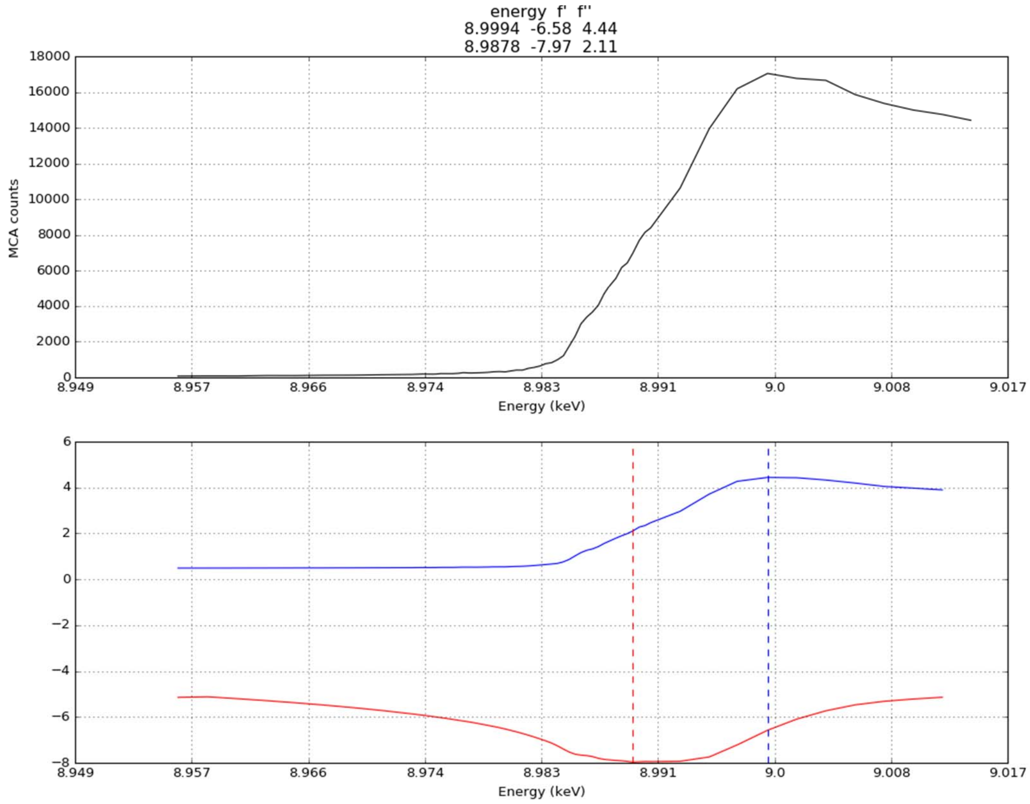

Figure S4 X-ray fluorescence scan for the GHK-GFP crystals around the copper absorption edge (between 8957 and 9010 eV). The experimental anomalous scattering for Cu is 4.44 é at 8999.4 eV (1.3777 Å).

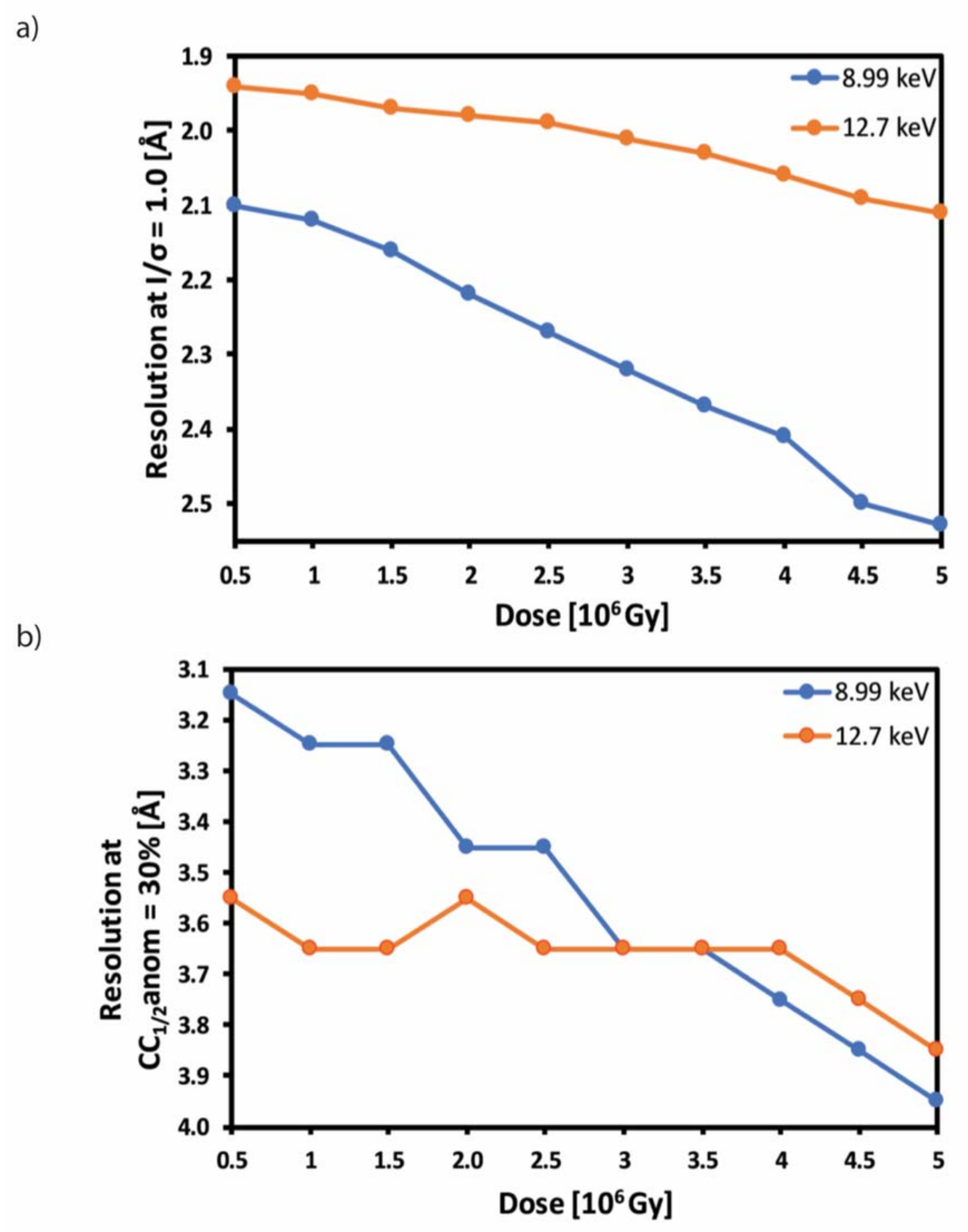

Figure S5 Global radiation damage as a function of resolution for the dose series acquired at 8.99 keV (blue) and 12.7 keV (orange). a) Decrease of the diffraction limit with increasing dose (cutoff I/ σ=1.0). b) Decrease of the anomalous signal with increasing dose (cutoff $CC_{1/2}$ anom=30%).

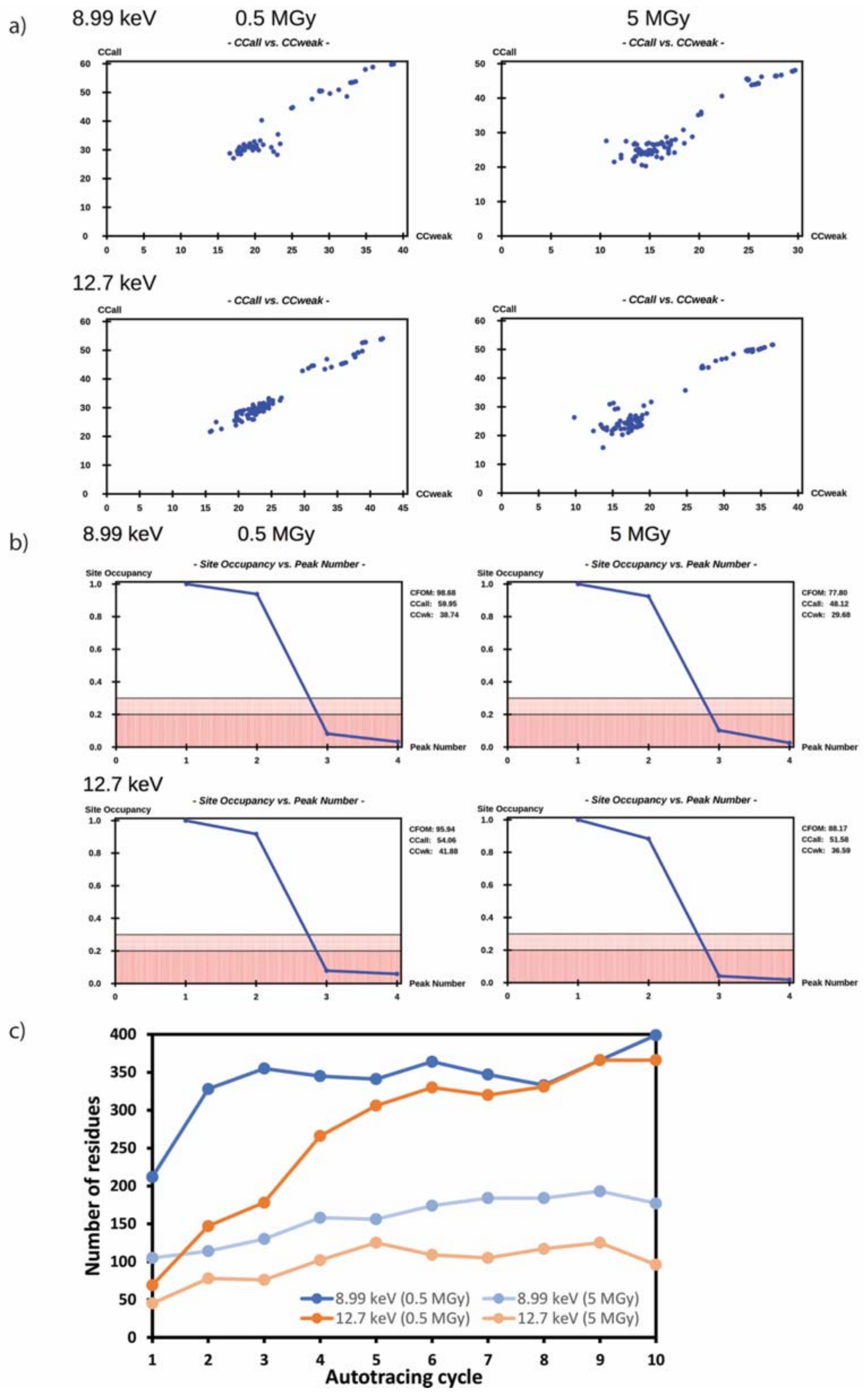

Figure S6 Influence of radiation damage on substructure determination and autotracing by *SHELXC/D/E*. a) *SHELXD* correlation coefficients for substructure determination of the first (0.5 MGy) and last (5 MGy) datasets of the dose series. c) Autotracing of the 0.5 MGy and 5 MGy datasets (acquired at the Cu absorption edge and at 12.7 keV) by *SHELXE*.

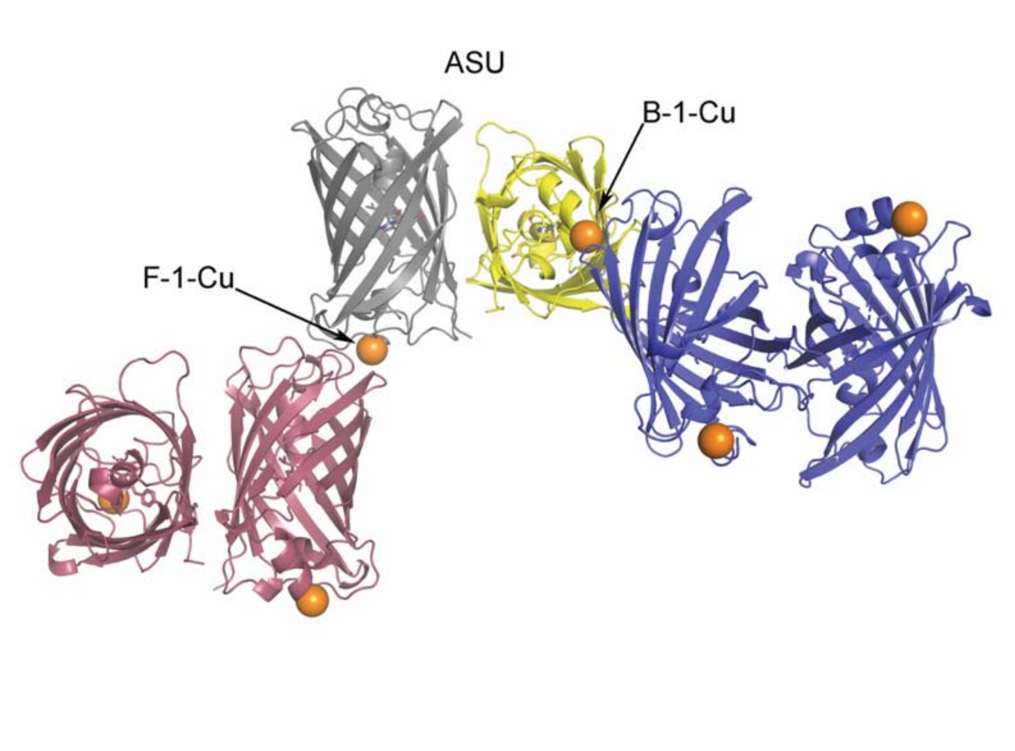

Figure S7 Copper binding sites between different GHK-GFP molecules within the ASU. The ASU dimer is coloured grey and yellow and symmetry related molecules coloured raspberry and blue.

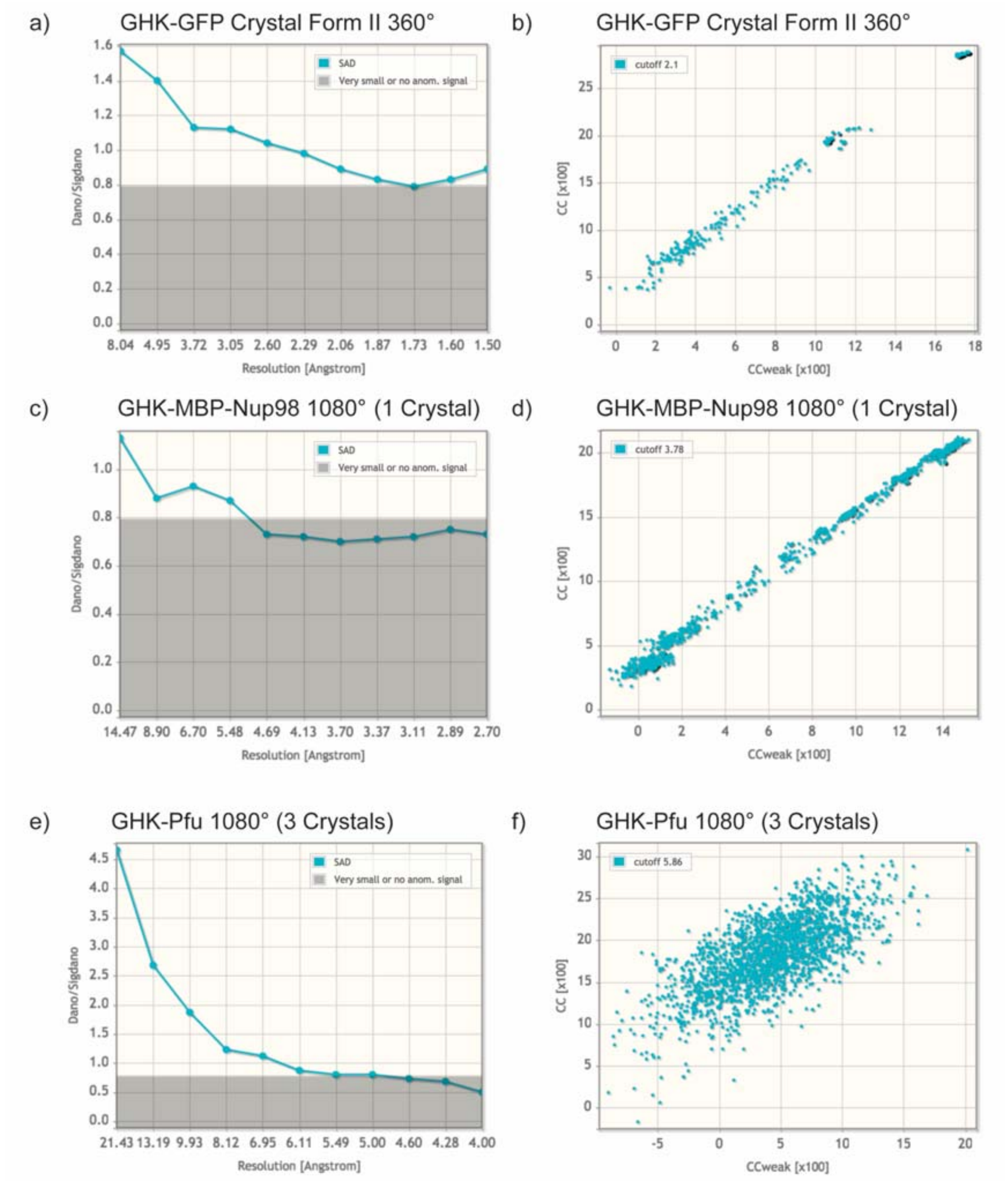

Figure S8 Output from *CRANK*2 pipeline run via *CCP*4 online (https://ccp4serv7.rc-harwell.ac.uk/ccp4online) for GHK-GFP crystal form II, GHK-MBP-Nup98 and GHK-Pfu. Panels a,c,e; Anomalous signal estimation output from *SHELXC*. Panels b,d,f; Profiles of *SHELXD* correlation coefficients (CC) for substructure determination. Results are shown for the datasets given in Table 1

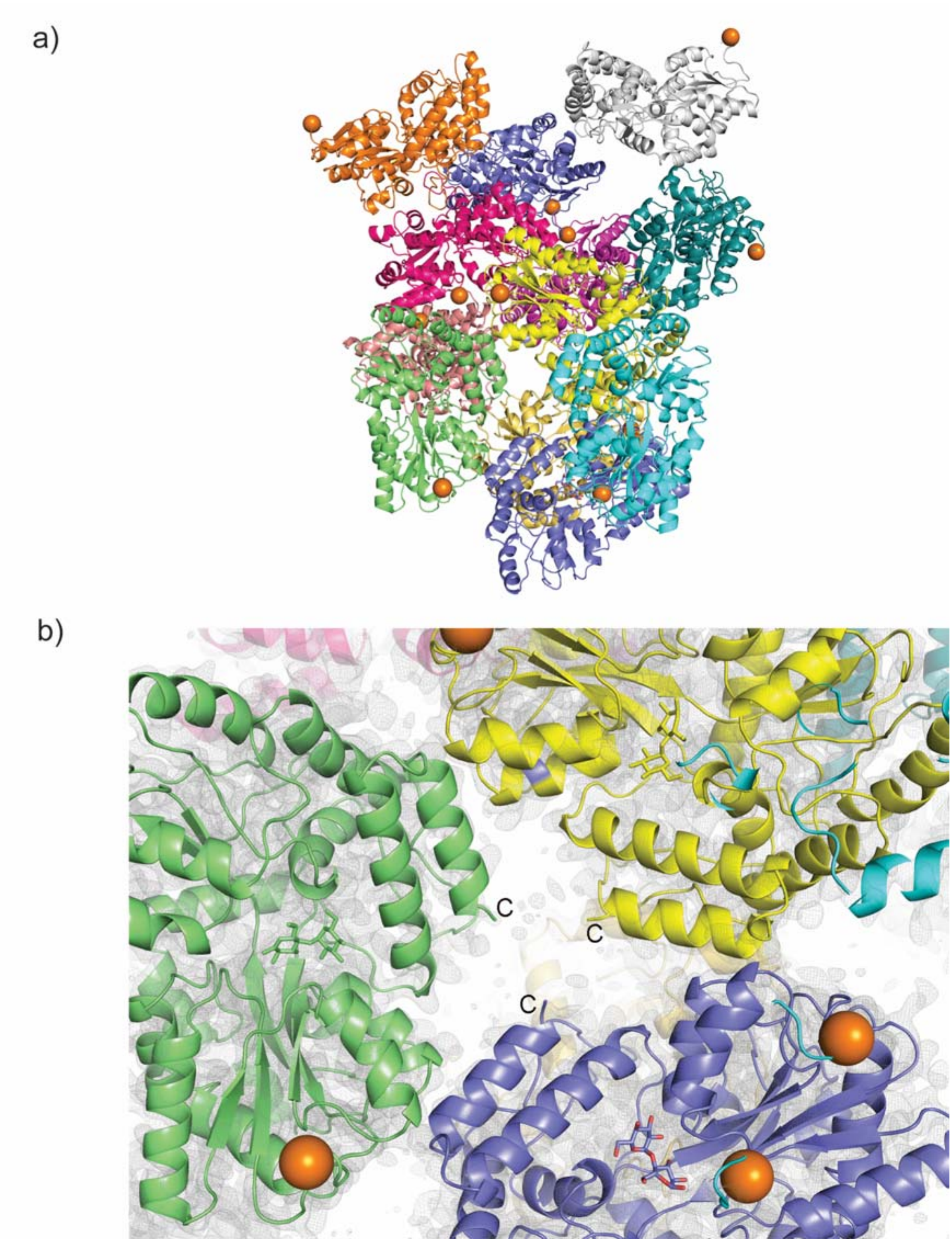

Figure S9 The ASU of GHK-MPB-Nup98 crystals is shown in a) as ribbons with each of the 12 molecules coloured differently, bound copper ions are shown as orange spheres. A close up of the C-terminal region from 3 molecules is shown in b) with the corresponding 2Fo-Fc map contoured at 1σ. It was not possible to trace the chain any further due to the flexibility of the Nup98 (1-29) sequence.

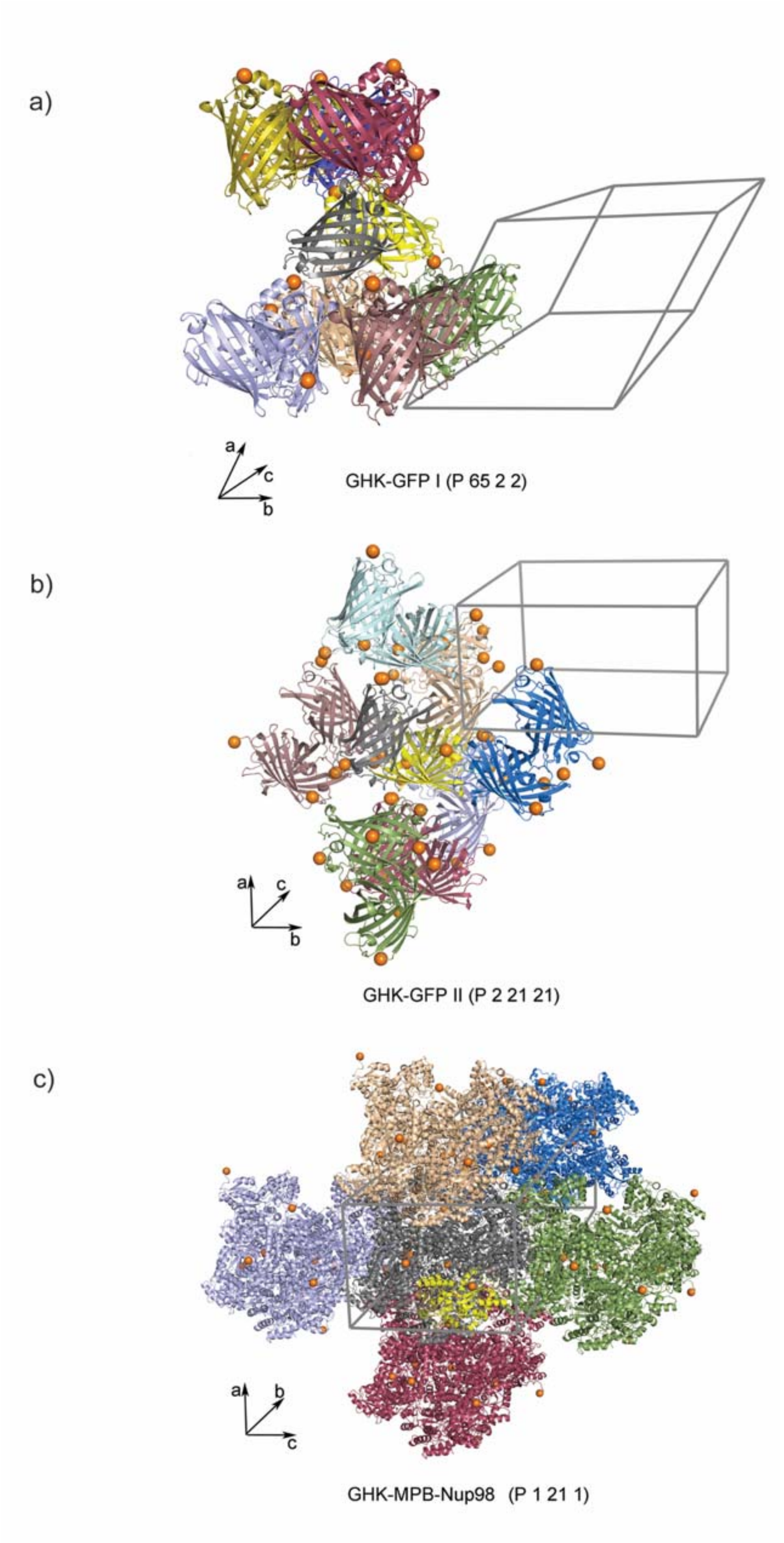

Figure S10 The crystal structures of a) GHK-GFP crystal form I, b) GHK-GFP crystal form II and c) GHK-MBP-Nup98. In each case the ASU is coloured grey with 1 molecule coloured yellow, symmery related ASU are shown in different colours and the unit cell is drawn. Bound copper ions are shown as orange spheres.

Table S1 Oligonucleotides sequences used

**Oligo**	**Sequence** (Vector Gibson assembly sites are in bold, GHK underlined)
GHK-GFP_F	5' **tcagactggtggc**ggccataaacgtggagaacaactgttcaccggggtg
GHK-GFP_R	5' **gtcgagcctac**gagctacgttatgccagcagcagtaacaaattccagtaggac
GHK-MBP-Nup98_F	5'**tcagactggtggc**ggccataaaaaaattgaagaaggtaaactggtaatctggattaacggcgat
GHK-MBP-Nup98_R	5' **gtcgagcctacgagc**tactgctgcggctgaccgaacagaccacc
GHK-Pfu_F	5‘ **tcagactggtggc**ggccataaaatgattttagatgtggattacataactgaagaagga
GHK-Pfu_R	5‘**gtcgagcctacgagc**tagtatctgaggtcttcctttctgtatccaaatcc
